# Weight gain in anorexia nervosa does not ameliorate the faecal microbiota, branched chain fatty acid profiles, and gastrointestinal complaints

**DOI:** 10.1038/srep26752

**Published:** 2016-05-27

**Authors:** Isabelle Mack, Ulrich Cuntz, Claudia Grämer, Sabrina Niedermaier, Charlotte Pohl, Andreas Schwiertz, Kurt Zimmermann, Stephan Zipfel, Paul Enck, John Penders

**Affiliations:** 1 Department of Psychosomatic Medicine and Psychotherapy, University Medical Hospital, Tübingen, Germany; 2 Klinik Roseneck, Center for Behavioral Medicine, Prien, Germany; 3 Institute of Microecology, Herborn, Germany; 4 SymbioPharm GmbH, Herborn, Germany; 5 Maastricht University Medical Center, NUTRIM School for Nutrition and Translational Research in Metabolism, Department of Medical Microbiology, Maastricht, The Netherlands

## Abstract

The gut microbiota not only influences host metabolism but can also affect brain function and behaviour through the microbiota-gut-brain axis. To explore the potential role of the intestinal microbiota in anorexia nervosa (AN), we comprehensively investigated the faecal microbiota and short-chain fatty acids in these patients before (n = 55) and after weight gain (n = 44) in comparison to normal-weight participants (NW, n = 55) along with dietary intake and gastrointestinal complaints. We show profound microbial perturbations in AN patients as compared to NW participants, with higher levels of mucin-degraders and members of *Clostridium* clusters I, XI and XVIII and reduced levels of the butyrate-producing *Roseburia spp.* Branched-chain fatty acid concentrations, being markers for protein fermentation, were elevated. Distinct perturbations in microbial community compositions were observed for individual restrictive and binge/purging AN-subtypes. Upon weight gain, microbial richness increased, however perturbations in intestinal microbiota and short chain fatty acid profiles in addition to several gastrointestinal symptoms did not recover. These insights provide new leads to modulate the intestinal microbiota in order to improve the outcomes of the standard therapy.

Anorexia nervosa (AN) is a serious eating disorder and has the highest mortality rate of all mental disorders and relapse is very common^1^,^2^. The treatment of AN is protracted and is based on psychotherapy applied in inpatient and outpatient settings^3^. Besides the difficulty of treatment acceptance by these patients^4^, gastrointestinal problems^5^ are complicating compliance. Only 50% of AN patients recover fully in the long-term^2^, so alternative therapies in addition to psychotherapy are needed to achieve better outcomes.

Besides psychological and environmental factors, physiological factors could be causative for AN^6^ – the gut microbiota could be one such factor. A disturbed gut microbiota has been linked to intestinal disorders such as inflammatory bowel disease^7^, and colon cancer and its association with atopic diseases, diabetes and metabolic syndrome underscore that the gut microbiota has a systemic impact on human health^8^. Furthermore, there is evidence that the gut microbiota influences brain function and behaviour of the host by communication via the gut-brain axis^9^.

Although, the composition of the intestinal microbiota plays an important role in gastrointestinal disorders and weight regulation^10^, the role of gut microbiota in undernourished adolescents and adults, including AN, has not been thoroughly investigated. Armougom *et al*.^11^, Million *et al*.^12^ and Morita *et al*.^13^ analysed a selected range of gut microorganisms in 9 AN, 15 AN and 25 AN patients, respectively. Armougom *et al*. reported that *Methonobrevibacter smithii*, a methane producing archaeon, was increased compared to normal weight and obese participants^11^. Morito *et al*.^13^ found that AN patients had lower amounts of total bacteria and obligate anaerobes as well as lower levels of acetate and propionate in their faeces when compared to normal weight participants. Another study investigated, in a small sample of AN patients before (n = 16) and after weight gain (n = 10), the relationship between mental health and gut microbiota. They found, that the microbial composition and diversity were associated with mental health^14^. Moreover, an explorative study found eleven new bacterial species in a stool sample of one AN patient^15^.

Extending this previous work, the aim of this study was to examine for the first time in a substantial number of participants i) to what extend the intestinal microbiota of AN patients is perturbed in comparison to NW participants and ii) whether these perturbations are recovered after weight gain and/or normalisation of eating behaviour. Besides the large number of participants, a special strength of this study is that we not only analysed the intestinal microbiota composition (using MiSeq technology with a deep sequencing depth of approx. 22.000 sequences/sample), but also i) the short chain fatty acids (SCFA) profiles (before and after weight gain) along with ii) dietary intake and iii) gastrointestinal complaints. Moreover, we were able to distinguish between differences in the intestinal microbiota community compositions in the different AN subtypes. These investigations could provide a basis for new leads for gut microbiota targeted-intervention aiming at alleviating gastrointestinal symptoms in AN patients and to support recovery and weight normalisation and possibly other forms of undernutrition.

## Results

### BMI differs by 6.3 points between AN patients before weight gain and NW participants, and by only 3.9 points after AN patient weight gain

Faecal samples were collected from 55 female AN patients at baseline (T1) and from 44 of these patients, a second sample was collected at the end (T2) of their impatient stay (44 paired samples) between September 2011 and October 2012. Between February 2013 and July 2013, stool samples of 55 healthy female NW participants were collected. The NW participants were matched for age and gender based on the respective data of the AN patients which had provided a T1 sample.

The average age was 23.8 ± 6.8 (min-max: 14–39) years in AN patients and 23.7 ± 6.7 (min-max: 14–38) years in NW participants. The mean BMI of NW participants was 21.6 ± 2.0 (min-max: 18–25) kg/m^2^ and of AN patients 15.3 ± 1.4 (min-max: 11.6–17.7) kg/m^2^ at T1 and 17.7 ± 1.4 (min-max: 13.9–21.8) kg/m^2^ at T2, respectively. Thus, the mean BMI in AN patients increased by 2.3 ± 1.2 (min-max: 0.0–4.9) BMI points during their inpatient stay of 14.0 ± 6.8 (min-max: 4–43) weeks. In the AN group, 39 patients belonged to the restrictive type and 16 patients to the binge/purging type. None of NW participants but six out of 55 AN patients reported using laxatives (2x Dulcolax®, 2x Movicol®, 1x lactulose and Dulcolax®, 1x Laxoberal®) before their inpatient stay with no specifications on frequency. One AN patient and none of NW participants reported regularly taking probiotics. There was no significant difference in the frequency of intake of yoghurt and quark between AN patients at T1 and NW participants (Supplement 1).

### Before weight gain, energy and macronutrient intake is low and fibre intake normal in AN patients whereas during weight gain the fat, energy and fibre intake is high

**Figure 1:**
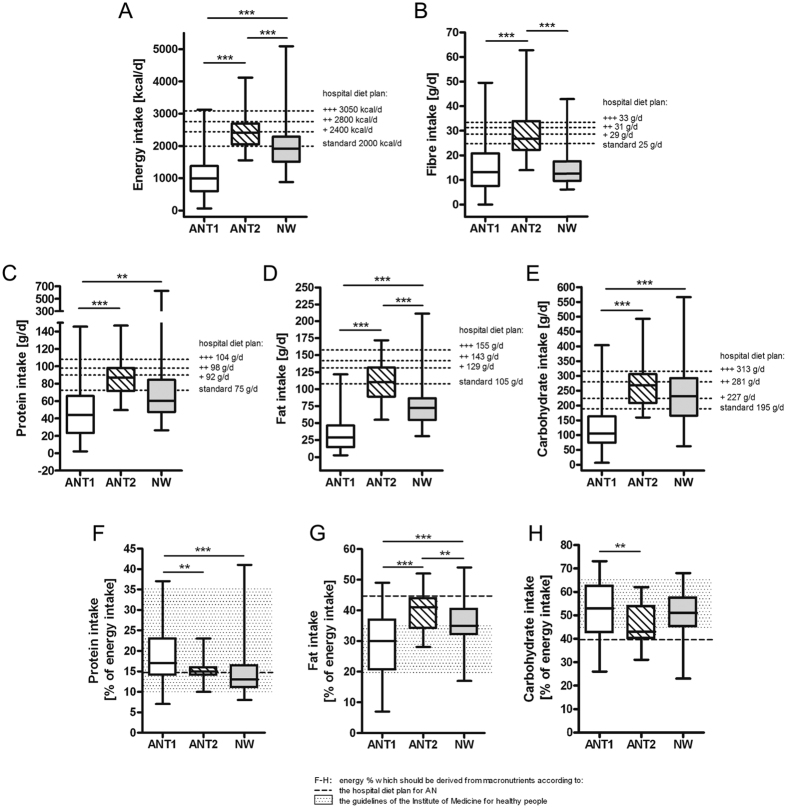
Energy and macronutrient intake of anorexia nervosa patients (AN) before (T1) and after (T2) weight gain in comparison to normal-weight participants (NW). The daily intakes of energy (**A**), fibre (sum of cellulose + non-cellulosic polysaccharides + lignin) (**B**), protein (**C**), fat (**D**) and carbohydrates (**E**), and the energy percentages derived from protein (**F**), fat (**G**) and carbohydrates (**H**) in the diet are shown for NW and AN at T1 and T2. A p-value < 0.01 was considered as statistically significant. **Indicates a p < 0.01, ***indicates a p < 0.001.

The energy and macronutrient intake of AN patients at T1 was low when compared to NW participants (Fig. 1A,C–E). AN patients avoided the intake of many foods but most of the patients reported eating fruit, vegetables and whole-wheat bread every day (Supplement 1). This explains the similar daily fibre intake of AN patients at T1 in comparison to NW participants (Fig. 1B). The percentage energy derived from macronutrients was within the guidelines of the Institute of Medicine (IOM^16^; Fig. 1F–H). During the inpatient stay, AN patients had to follow the strict diet plans from the hospital which resulted in higher energy, fat and fibre intake when compared to NW participants (Fig. 1A,B,D). Accordingly, there was a change in the frequency of intake of foods but some were still avoided (Supplement 1).

### Upper and lower gastrointestinal (GI) symptoms are frequent in AN patients, and some improve in the course of weight gain whereas others do not

**Figure 2:**
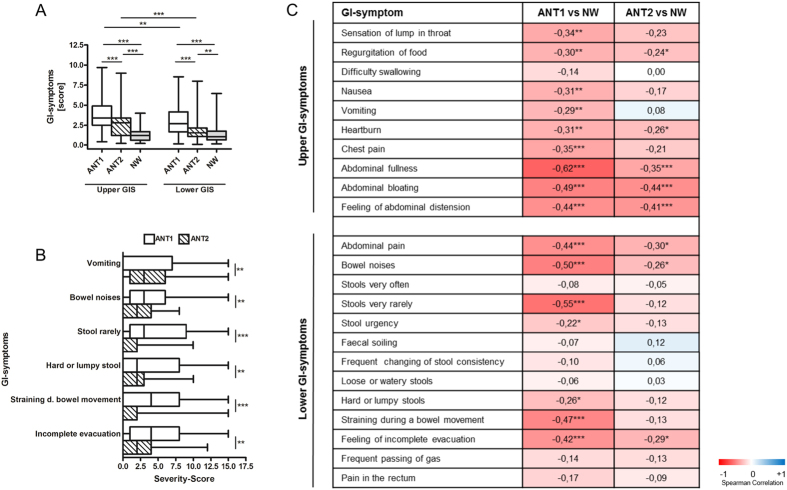
Severity scores for Gastrointestinal symptoms (GIS) in anorexia nervosa patients (AN) before (T1) and after (T2) weight gain and in comparison to normal-weight participants (NW). (**A**) Upper GIS and lower GIS for all groups are presented. (**B**) Significant changes of GIS between T1 and T2 in AN are given. A complete overview of GIS between T1 and T2 in AN is given in Supplement 2. (**C**) The heatmap shows Spearman correlations between ANT1 versus NW and between ANT2 and NW, respectively for GIS. Negative correlations (in red) indicate increased Severity-Scores of the specific symptoms in AN compared to NW. Positive correlations (in blue) indicate decreased Severity-Scores of the specific symptoms in AN compared to NW. All p-values were false discovery rate (FDR)-adjusted. A FDR < 0.05 was considered as statistically significant. **Indicates a FDR < 0.01 and ***a FDR < 0.001.

Lower and especially upper GI symptoms were common in AN patients at T1 when compared to NW participants (Fig. 2A,C). Overall symptom scores of upper and lower GI symptoms improved at T2 (Fig. 2A), while the majority of individual upper GI-symptoms did not change significantly with the exception of (self-induced) vomiting (Fig. 2B,C, Supplement 2). The severity of the symptoms regurgitation of food, heartburn, abdominal fullness, abdominal bloating and feeling of abdominal distension did not ameliorate significantly from T1 to T2 (Fig. 2C, Supplement 2). In contrast, many symptoms of the lower GI improved (Fig. 2B). However, abdominal pain, bowel noises and feeling of incomplete evacuation were still more severe in AN patients at T2 than in NW participants (Fig. 2C).

### Sequencing

A total of 22,133,491 V4 reads were generated. After trimming, quality filtering, removal of potential chimeric reads and de-multiplexing, 9,674,034 sequences were retained for downstream analysis and clustered into 14,433 operational taxonomic units (OTUs). One NW participant’s sample failed during sequencing and was excluded from subsequent analysis. For the remaining samples, the number of sequences per sample ranged from 26,190 to 96,851 (median 62,895). To assess the reproducibility of DNA-isolation, amplicon library preparation and sequencing, we included duplicate samples from three participants. Beta-diversity distances between these duplicates were very low (Weighted Unifrac distance ranged from 0.03–0.11, Bray-Curtis distance ranged from 0.11–0.21) indicating highly reproducible results.

### The intestinal microbiota composition changes in AN in the course of weight gain

**Figure 3:**
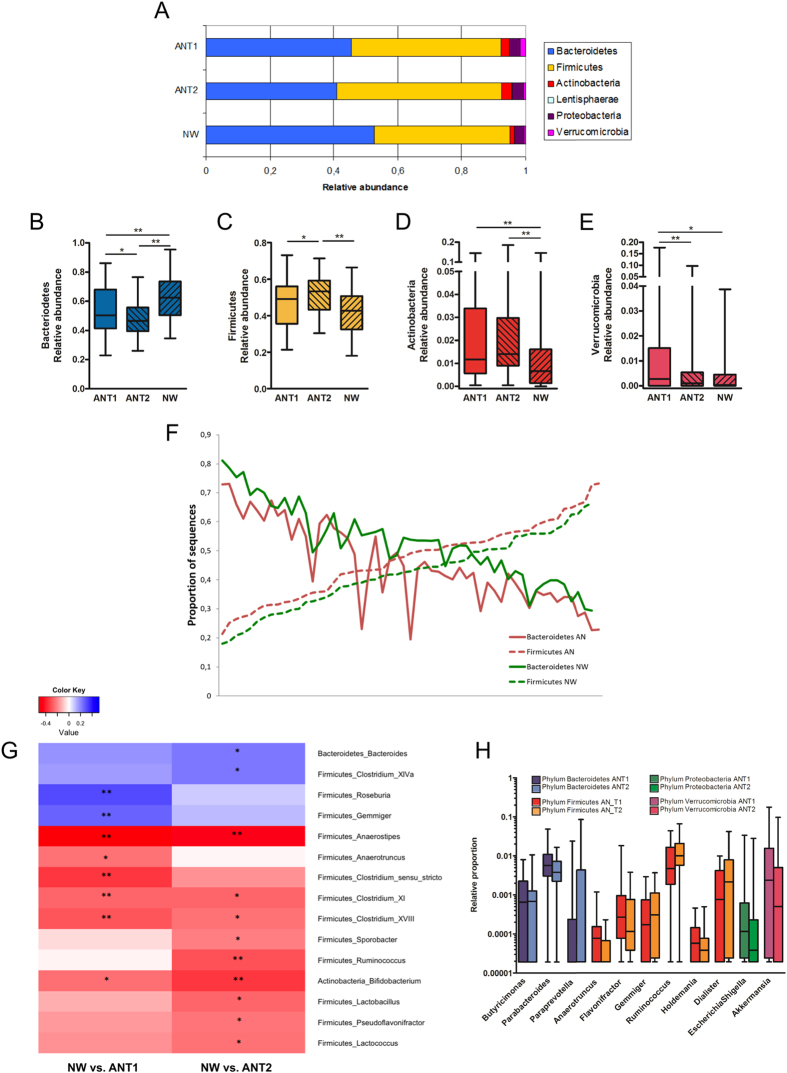
Taxonomy at phylum and genus level for anorexia nervosa patients (AN) before (T1) and after (T2) weight gain compared to normal-weight participants (NW). (**A**) An overview of the mean relative abundance at phylum level is given for taxa being present in at least 25% of all participants. Significant differences between the groups at phylum level are presented separately in box-whiskers for Bacterioidetes (**B**), Firmicutes (**C**), Actinobacteria (**D**) and Verrucomicrobia (**E**). (**F**) Relative abundance of Firmicutes and Bacteroidetes per AN and NW are shown. Subjects are sorted according to the relative abundance of Firmicutes. (**G**) The heatmap shows significant Spearman correlations between ANT1 versus NW and between ANT2 and NW, respectively at genus level. Positive correlations (in blue) indicate decreased levels of the specific taxon in AN compared to NW. Negative correlations (in red) indicate increased levels in AN compared to NW. (**H**) Significant differences within AN from T1 to T2 are presented. (**B**–**E**,**G,H**) The p-values were false discovery rate (FDR)-adjusted. In order to account for the beta error a FDR < 0.15 was considered as statistically significant. A FDR < 0.15 was found for all displayed taxa. *Indicates a FDR < 0.15 and **a FDR < 0.05.

Bacteroidetes, Firmicutes, and, to a lesser extent, Actinobacteria, Proteobacteria and Verrucomicrobia were the dominant phyla in both AN patients and NW participants (Fig. 3A). AN patient and NW participant samples showed profound differences with respect to the relative abundance of these bacterial phyla. Compared to NW participants, the relative abundance of Bacteroidetes was significantly lower in AN patients at baseline and decreased even further after weight gain (Fig. 3B). In contrast, the relative abundance of Firmicutes significantly increased in AN patients from T1 to T2. As a consequence, at T2, the relative abundance was significantly higher in AN patients when compared to NW participants (Fig. 3C). Figure 3F depicts the relative abundance of both Firmicutes and Bacteroidetes per study subject at baseline, highlighting the lower Bacteroidetes to Firmicutes ratio in AN patients.

Actinobacteria levels were high in AN patients at T1 and T2 when compared to NW participants (Fig. 3D) whereas Verrucomicrobia relative abundance was high in AN patients at T1, but decreased after weight gain (Fig. 3E).

The majority of significant differences in the relative abundance of bacterial genera between AN patients and NW participants (Fig. 3G) as well as in AN patients over time (Fig. 3H) were found within the phylum of Firmicutes.

Archaea, with the main genus *Methanobrevibacter*, were detected in 22% of ANT1 patients, in 14% of ANT2 patients and in 15% of NW participants. When analysing the data for the participants harbouring this archaeon, the relative abundance was statistically significantly higher in AN patients at T1 when compared to NW participants (% relative abundances: ANT1: 0.10 [0.05–0.14], NW: 0.01 [0.04–0.40], p = 0.004). Within AN patients, the relative abundance of *Methanobrevibacter* decreased in 7 and increased in 3 out of 10 patients from T1 to T2.

**Figure 4:**
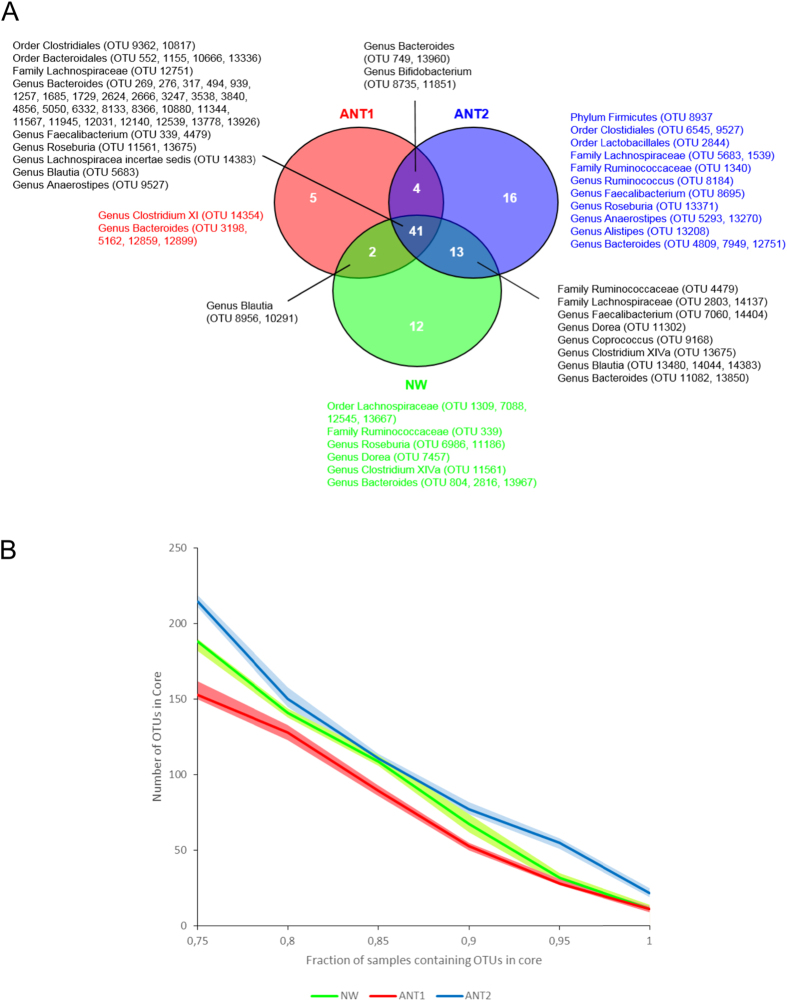
The core microbiome of anorexia nervosa patients (AN) before (T1) and after (T2) weight gain compared to normal-weight participants (NW). (**A**) The Venn diagram shows the core microbiome of AN at T1 and T2 and of NW. For each of the groups the core microbiome was defined as the OTUs that were observed in at least 90% of the participants. Bacterial taxa belonging only to the core of ANT1, ANT2 or NW are listed in respectively red, blue and green. (**B**) The fraction of samples in the core microbiome of AN at T1 and T2 and of NW are presented. The number of OTUs part of the core microbiome defined as the OTUs that were observed in a minimal fraction of subjects ranging from 75% to 100%. Lines represent the mean number of OTUs in the core (based upon 10 rarefactions), surrounding areas represent the 95% confidence intervals.

The core microbiome (Fig. 4A), defined as a minimum of 90% of samples containing a specific OTU, contained 41 OTUs that were shared by all three groups. NW participants shared 54 OTUs in their core with ANT2 patients of which 13 OTUs were lacking at T1. Among these were OTUs belonging to the genera *Coprococcus, Dorea* and *Clostridium cluster XIVa*, all lacking in the core of ANT1 patients. *Clostridium cluster XI* was only present in the core of ANT1 patients. There were also 4 OTUs, which were shared only between ANT1 and ANT2 patients with two of these OTUs belonging to the genus *Bifidobacterium*, whereas bifidobacteria did not occur in the core of NW participants. Moreover, when looking at the core microbiome over a window of different maximum fractions of samples that should be positive for an OTU to be included in the core, we found the lowest core size for AN patients at T1 and the highest for AN patients at T2 (Fig. 4B).

### Microbial diversity and richness are normal in AN patients before weight gain and increase in the course of weight gain

**Figure 5:**
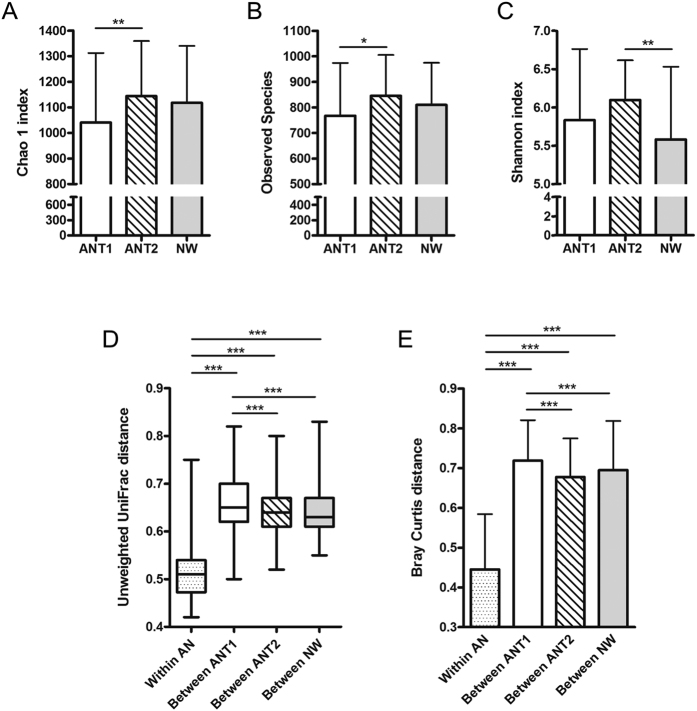
Alpha and beta diversity metrics in anorexia nervosa patients (AN) before (T1) and after (T2) weight gain compared to normal-weight participants (NW). The number of observed OTUs (**A**), Chao 1 index (**B**) and Shannon index (**C**) for all groups are shown. The p-values of all metrics were Bonferroni adjusted. A p < 0.05 was considered as statistically significant. *Indicates a p < 0.05 and **indicates a p < 0.01. Unweighted UniFrac (**D**) and Bray Curtis (**E**) distances were calculated within AN subjects (average pairwise distance in microbiota composition between samples of the same AN patient at T1 and T2) and between subjects (average pairwise distance between samples of different subjects) for the AN groups at T1 and T2 and for the NW group, respectively. A p-value < 0.001 was considered as statistically significant. ***Indicates a p < 0.001.

An overview of the alpha diversity metrics and significant differences between the groups is given in Fig. 5A–C. The observed number of OTUs increased significantly in AN patients from T1 to T2 but no differences to NW participants were observed (Fig. 5A). In concordance, estimated microbial richness measured with the Chao1 index showed the same significant differences between the groups (Fig. 5B). The biodiversity, as measured with the Shannon index, was significantly increased in ANT2 patients in comparison to NW participants (Fig. 5C).

### The microbial community structure is related to disease status (AN patients versus NW participants) but the latter is not linked to enterotypes

The dissimilarity in the microbial community composition (beta-diversity) of stool samples was assessed using the (un) weighted UniFrac and Bray-Curtis distances. The intra-individual dissimilarity in microbial composition in AN subjects (average pairwise distance between the T1 and T2 samples of the same AN subject) was significantly lower than the dissimilarity between subjects (average pairwise distance between samples of different subjects) in the respective groups, ANT1, ANT2 and NW (Fig. 5D,E). This indicates that the faecal microbiota composition of AN patients after weight gain is still more similar to the microbiota composition of the patients’ own faecal samples at admission than it is to the microbiota composition of other individuals.

The phylogenetic (Unifrac) distances between AN patients at baseline were larger than distances between AN patients after weight gain as well as between NW participants. Following weight gain (T2), the distances between AN patients decreased and were comparable to those observed in NW participants.

**Figure 6:**
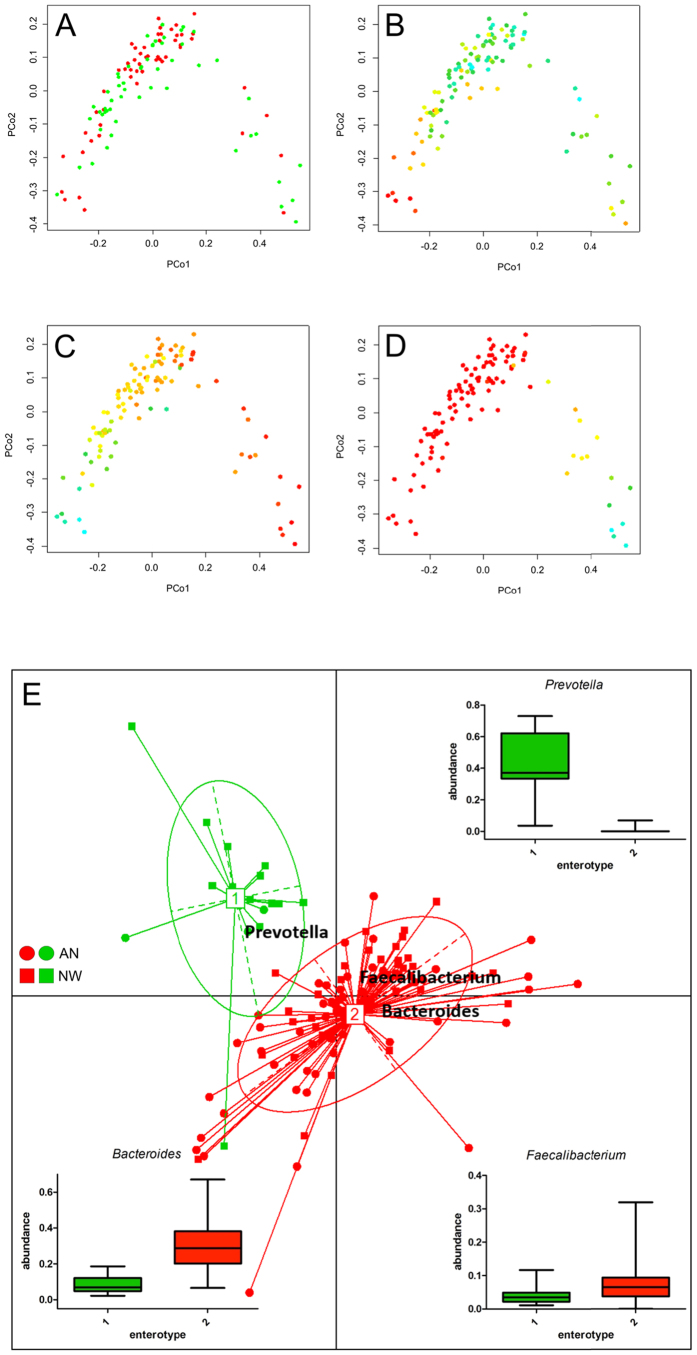
Principal Coordinate Analyses (PCoAs) based on Bray-Curtis distance and assortment of gut microbial communities into enterotypes for anorexia nervosa patients before weight gain (ANT1) and normal-weight participants (NW). (**A**) PCoA, colored according to NW (green), ANT1 (red), (**B**) PCoA, colored according to microbial richness (Chao1), (**C**) PCoA, colored according to Bacteroides abundance, (**D**) PCoA, colored according to Prevotella abundance. Plots (**B**–**D**) are colored using a gradient ranging from low (red) to high (cyan-blue). (**E**) Shown are between-class analysis visualizations of enterotypes, as identified by PAM clustering, with closed dots and squares representing individual AN patients or NW participants, respectively and numbered white rectangles marking the center of each enterotype. The box-whiskers present the relative abundances of the three bacterial taxa that are mainly driving the separation of the enterotypes.

**Figure 7:**
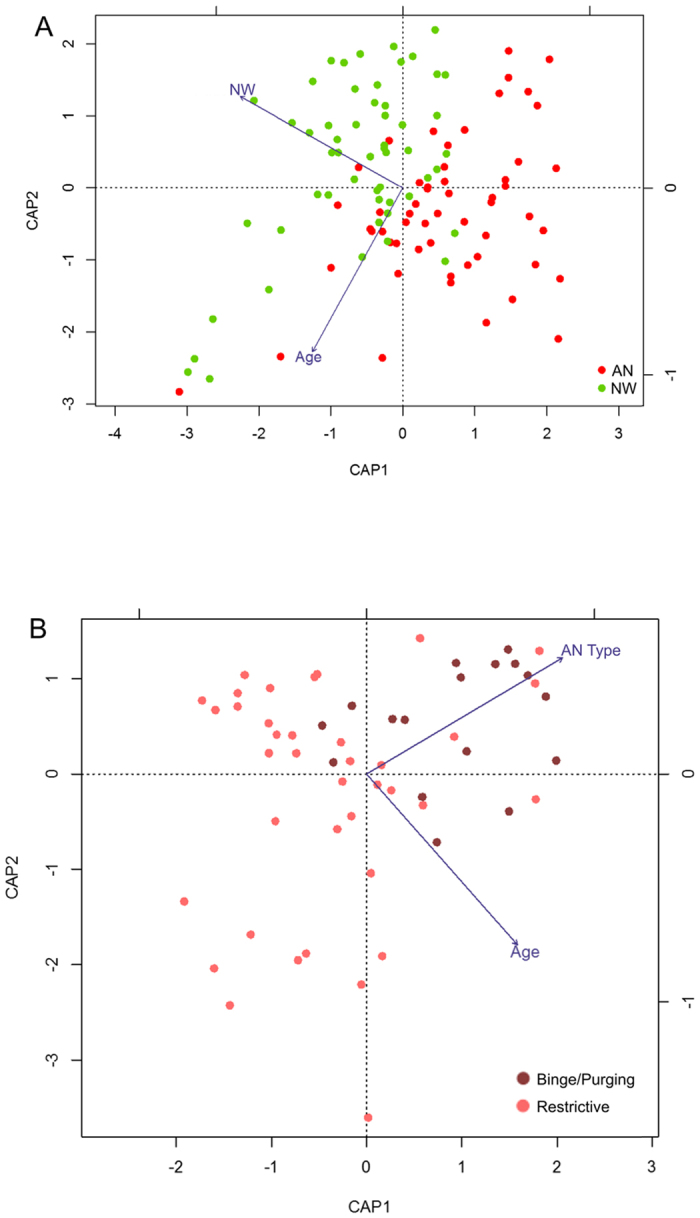
Distance-based Redundancy Analysis (db-RDA) plot showing the relationship of age and disease status to the microbial community structure. The plots represents a dbRDA ordination based upon the Bray-Curtis distance including (**A**) anorexia nervosa patients (AN) at baseline (T1) and normal-weight participants and with age and disease status as explanatory variables or (**B**) AN at baseline at T1 and with age and disease status, gastrointestinal symptoms, BMI, total energy intake and fibre, fat and protein intake as explanatory variables. Age and anorexia subtype were the only significant explanatory variables. Binge purging = Binge/Purging AN-type, Restrictive = Restrictive AN-type.

Principal coordinate analysis (PCoA) based on Bray-Curtis dissimilarities showed that variation explained by the first two principal coordinates was not related to disease status (Fig. 6A). Instead the relative abundance in *Bacteroides* and *Prevotella* (first coordinate, Fig. 6C,D) and microbial richness, (second coordinate, Fig. 6B) were driving separation. Similarly, PAM clustering assorted the gut microbial communities into two enterotypes, driven by *Prevotella, Bacteroides* and *Faecalibacterium*, which did not show a correlation with disease status. (Fig. 6E). To determine, whether disease status or other variables had an additional effect on the gut microbiota community structure, we performed distance-based redundancy analysis (dbRDA); a constrained ordination technique. Constrained ordination techniques attempt to explain differences in microbial composition between samples by differences in explanatory variables (e.g. disease status). In db-RDA, the information from explanatory variables is combined with the eigenvalues obtained from PCoA. The disease status (ANT1 patients versus NW participants, p = 0.011) and to a lesser extent, age (p = 0.062, not significant) showed a relationship with the microbial community structure (Fig. 7A), whereas energy and macronutrient intake and upper and lower GI symptoms did not. Moreover, when db-RDA was applied on the patient samples (ANT1) only, it was found that the AN subtypes, displaying the restrictive versus the binge-purging type, had an additional effect on the microbial community structure (p = 0.008; Fig. 7B).

### Total SCFA levels are normal in AN patients whereas the concentrations and proportions of branched chain fatty acids (BCFA) are increased before and after weight gain

Finally, SCFA were analysed in the stool samples, which are end products of fermentation in the colon. SCFA mainly represent products of carbohydrate fermentation whereas BCFA (consisting of isobutyrate and isovalerate) are products of protein fermentation^17^,^18^.

The SCFA concentrations, proportions and statistics are presented in Table 1. The concentrations of total SCFA, acetate, propionate and butyrate were comparable between AN patients and NW participants, but butyrate proportions were slightly decreased in ANT1 patients versus NW participants. In AN patients at T1 and at T2 in comparison to NW participants, total BCFA and valerate concentrations were increased. In relation to the total SCFA, this resulted in smaller proportions of butyrate and propionate and larger proportion of total BCFA and valerate in AN patients at T1 when compared to NW participants. When calculating a ratio of the sum of acetate, butyrate and propionate divided by the sum of valerate, isovalerate and isobutyrate the median values of AN patients at T1 (11.9 [8.1–17.1]) were lower when compared to T2 (13.6 [9.7–18.3], p < 0.05) but both AN ratios were substantially lower when compared to NW participants (21.2 [15.3–26.7], p < 0.001).

Finally, we examined to what extent bacterial genera (found at least in 25% of our participants) correlated with SCFAs. We did the analyses separately for all groups (AN patients at T1 and T2 and NW participants) in order to prevent confounding by disease status (Supplement 3). It is noteworthy that *Roseburia* was the only genus that correlated with butyrate in all three groups although, in NW participants, the correlation was not evident after FDR adjustment.

### AN patients with a history of laxative use before their inpatient stay did not substantially influence the reported results

The use of laxatives is common in a subpopulation of AN patients, which is why we initially did not exclude the patients (n = 6) who reported a history of laxative use (+lax AN) before their inpatient stay. We found that the observed number of OTUs and the Chao1 index were significantly lower in +lax AN patients in comparison to patients without a history of laxative use (−lax AN; Supplement 4A). A PCoA plot of Bray-Curtis dissimilarities shows that 3 out of the 6 laxative users tend to group at the border of the PCoA (Supplement 4B).

Therefore, we performed sensitivity analysis for the alpha diversity indices (Supplement 4C) and, taxonomy at phylum (Supplement 4D) and genus level (Supplement 4E) in NW participants versus −lax ANT1 patients, thus excluding patients with a history of laxative use. Results with respect to the alpha diversity indices remained similar to the main analysis. At the taxonomic level, the relative abundance of a few additional taxa differed significantly between AN patients and NW participants, when excluding +lax AN patients. At phylum level a difference of Firmicutes and at genus level differences of *Bacteroides spp*. and *Akkermansia spp.* between NW participants and −lax ANT1 patients became significant. For all these differences, a (non-significant) trend was already apparent in the main analysis.

## Discussion

Our study reports - for the first time - the combined intestinal microbiota and SCFA profiles in a substantial number of AN patients ahead of and after weight gain in comparison to normal-weight participants matched for age and gender, along with dietary intake and gastrointestinal complaints. The weight gain of the patients was high with 2.3 ± 1.2 BMI within 14.0 ± 6.8 weeks.

With regard to weight gain, the literature focuses mainly on the role of the gut microbiota in obesity^7^. Nevertheless, important work regarding the role of gut microbiota and undernutrition in children has been conducted^19^,^20^. However, there is a lack of human studies investigating the gut microbiota of malnourished adults and adolescents, including AN patients. For the latter, first important work has recently been conducted as reviewed extensively in the introduction. We found i) profound differences in the microbial composition between AN patients before weight gain and NW participants and ii) that the intestinal microbiota of AN patients after substantial weight gain and NW participants did not converge. Metabolomics of AN patients revealed a similar pattern where concentrations of many metabolites in controls differed even more strongly from those of AN patients after short-term weight gain than before weight gain^21^. Additionally, our AN patient stool samples contained increased levels of BCFA, markers for protein fermentation, before and after weight gain when compared to NW participants. Although the GI symptoms overall improved in AN patients in the course of weight gain, a period of more than a mean of 3 month impatient treatment was insufficient to satisfactorily abolish GI symptoms.

Our dietary assessment revealed, that the amount of dietary fibre can serve as a basic substrate for the gut microbiota in AN patients. Nevertheless, there are also other dietary substrates escaping the digestive tract – non digestible oligosaccharides (e.g. inulin and oligofructose), proteins, fats, even di- and mono-saccharides and especially resistant starch^22^,^23^. Due to the overall decreased macronutrient and energy intake of AN patients at T1 it is likely that, except for fibre, all other dietary sources escaping digestion reached the colon in much smaller amount than in NW participants.

As such, fibre and endogenous host and microbe-derived proteins and carbohydrates (e.g. mucins being glycoproteins) are most likely the main components nourishing the gut microbiota. Since constipation-associated symptoms (infrequent and rare stools, hard or lumpy stool, straining during a bowel movement) were higher in AN patients than in NW participants, indicating slow colonic transit times, this may offer an additional advantage for some microbes (e.g. mucin degrader^24^).

In general, a lower bacterial diversity is considered detrimental for (intestinal) health as it has been linked to many other diseases^8^. In contrast to findings in malnourished Bangladeshi children^20^,^25^ or neonatal mice subjected to reduced versus full lactation^26^ and a small sample of AN versus healthy patients^14^, microbial richness and evenness was similar in AN patients and NW participants. Therefore, we speculate that the normal intake of fibre and the normal distribution of the percentage of energy derived from macronutrients are critical for the inconspicuous alpha-diversity in AN patients that we observed.

At taxonomy level, we observed a reduced relative abundance of Bacteroidetes at T1. Accordingly, Bacteroidetes were also decreased in undernourished neonatal mice^26^, and children from Bangladesh^25^.

In line with our assumptions based on GI-symptoms and dietary assessment, we found - at genus level - lower levels of carbohydrate utilising taxa and higher relative abundances of mucin and protein degrading taxa. In detail, the carbohydrate degrading *Roseburia spp.*^27^ and *Gemminger spp.*^28^ were decreased. *Roseburia*, a key butyrate producer^18^, was also the only genus which correlated positively with butyrate for all three groups independently which may reflect its important role in this function and explain decreased butyrate proportions in AN patients before weight gain.

Mucins are important as enterocyte protective barrier against pathogens but also against (bio)chemical and physical damage. Several mucin-degrading bacteria (Verrucomicrobia^29^, *Bifidobacteria*^29^, *Anaerotruncus*^30^) were higher in this nutrient-deprived ecosystem when compared to NW participants. Our findings are in line with the literature where Verrucomicrobia (mainly *Akkermansia muciniphila*) exhibited an inverse relationship with body weight in mice^31^ and humans^32^,^33^. Moreover, Verrucomicrobia were highly increased in undernourished neonatal mice^26^, fasted hamsters^34^, and fasted humans^35^. Whereas Verrucomicrobia are typical mucin-degraders^29^, *Bifidobacteria*, being well-known representatives of the phylum Actinobacteria, are able to convert diet and host derived carbohydrates^29^. In malnourished children from Bangladesh^25^ and in AN patients from Japan^13^, no changes were observed in Actinobacteria whereas in fasting humans *Bifidobacteria* were also increased^35^. Notably, *Bifidobacteria* were in the core microbiome of AN patients but not in NW participants, implying that the vast majority of AN patients had specific *bifidobacterial* taxa in their microbiome in contrast to the healthy NW participants. *Anaerotruncus spp*., belonging to the phylum Firmicutes, were also increased in AN patients ahead of weight gain and are likely to be involved in mucin degradation; e.g., *Anaerotruncus colihominis* encodes the Nan cluster, which is important for mucin degradation^30^. Finally, some genera harbouring species involved in protein degradation (*clostridium* cluster I (sensu stricto) and cluster XI) were increased in AN patients at T1 when compared to NW participants^36^.

Interestingly, the archaeon *Methanobrevibacter* was detected in less than 20% of our participants, but in those where it was detected, higher relative abundances were found in AN patients before weight gain when compared to NW participants. This is in line with the findings of Armougom *et al*.^11^ who analysed n = 9 AN patients, and Million *et al*. showing a weak negative correlation (r = −0.20) between the BMI values and *M. smithii* concentrations in participants harbouring this archaeon^12^. In patients carrying *M. smithii*, this taxon might contribute to energy exploitation and may play a role in constipation^11^.

Coinciding with the differences in taxonomy between AN patients before weight gain and NW participants, our constrained analyses revealed differences between these groups, but also between restrictive AN and binge-purging AN patients. This finding was not surprising since the patients with binge/purging type have another eating/nutrition behaviour, characterized by occasionally eating relatively large amounts often followed by vomiting.

In AN patients, the observed increased BCFA and valerate concentrations and proportions, being markers for protein fermentation^17^,^18^ are likely to be a result of endogenous sources (e.g. bacterial secretions and lysis products, mucosal and bacterial cells). Morito *et al*.^13^, found decreased acetate and propionate concentrations in AN patients, whereas in our study acetate and propionate levels were not altered. An explanation could be a difference in the intestinal microbiota composition between European versus Japanese AN patients due to differences in diet (e.g. fish consumption, other starch and vegetable sources) and lifestyle. Nevertheless, in agreement with our results, Morito *et al*.^13^ also showed a clear trend for an increased BCFA concentration in AN patients, albeit not statistically significant and probably due to their small sample size. Besides BCFA, other products including phenols and indoles (both co-carcinogens), ammonia (mutagen/cellular poison), amines (neurotransmitter/mutagen precursors), HS^−^ and thiols (both cellular toxins)^18^ are formed during protein fermentation. These are detrimental metabolites that have the potential to negatively impact on the host gut physiology and motility as well as on psychology (e.g. depression), the latter via the gut-brain axis^17^,^37^. It is tempting to speculate that these metabolites could also contribute to a perpetuation of the condition and consequences of poor nutrition.

During weight gain, AN patients were subjected to a high-fat, high fibre and high-energy diet and the gastrointestinal symptoms partially improved, especially symptoms linked to slow colonic transit. Therefore, we assumed that the excess supply of dietary derived substrates would offer beneficial conditions for carbohydrate utilizing taxa and this, together with shorter transit times, would lead to a shift in mucin-degrading taxa.

Species richness increased from T1 to T2, although there was no difference when compared to NW participants. However, the diversity of microbes (Shannon index) was higher after weight gain in AN patients when compared to NW participants, which may be a result of increased evenness. Kleiman *et al*. did not see an increase of alpha diversity from T1 to T2 (n = 10) or in comparison to NW participants (n = 12)^14^.

Overall, there were clear alterations in taxonomy for the whole group. The phylum Bacteroidetes decreased after weight gain, this time also *Bacteroides spp*. were significantly reduced when compared to NW participants. *Parabacteroides spp.* decreased from T1 to T2. In contrast, Firmicutes increased, being significantly higher than in NW participants at T2. As expected, there were no differences for the carbohydrate utilising *Roseburia spp.* and *Gemmiger spp.* between AN patients and NW participants anymore, which may explain the normalized butyrate proportion. Moreover, *Ruminococcus* increased in the course of weight gain in AN patients, which may also reflect the increased amount of fibre and resistant starch in the diet^38^,^39^.

The decrease of Bacteroidetes and increase of Firmicutes may be a result of the high-fat, high-caloric diet, since similar proportions of these phyla have been described for obese mice^40^,^41^. However, for humans the situation is less clear^42^. One study found that a short-term high-calorie diet lead to a decrease of Bacteroidetes and an increase of Firmicutes, respectively^43^. In mice, it has been even shown that this relation in phyla distribution resulted in an increase of host energy-harvest from the diet^44^. We cannot exclude an increased energy-yield from the diet in our weight gaining AN patients but the stool energy loss (measured as the concentration of total SCFA) was normal.

As assumed, there was a shift in mucin-degrading taxa. Verrucomicrobia decreased to levels similar in NW participants, and for *Akkermansia spp*. a decrease from T1 to T2 was observed. The relative abundances of *Anaerotruncus spp.* decreased in the course of weight gain when compared to NW participants. In contrast, the relative abundance of *Actinobacteria* remained high in AN patients after weight gain, the difference in *Bifidobacteria* became even larger when compared to NW participants. This suggests that the already established *Bifidobacteria* may have benefitted from the diet-derived carbohydrates.

Finally, the concentrations and proportions of BCFA and valerate were still elevated in AN patients after weight gain, paralleled by increased relative abundances of microbes (species of *clostridium* cluster XI and *Lactobacillus*)^23^,^36^ involved in protein fermentation when compared to NW participants. Thus, protein fermentation still appears to play a superior role in AN after weight gain. Since AN patients still have gastrointestinal complaints, the gastrointestinal physiology appears not to be restored after weight gain. Therefore, endogenous protein sources may still be elevated despite the increase of protein originating from the diet.

From an evolutionary viewpoint, an intact intestinal microbiota-host symbiosis must be extremely important in general and particularly in periods of undernutrition to maintain a healthy body in order to exploit as much energy as possible when plenty of food is available. Nevertheless, the critical questions may be i) at which point will chronic undernutrition lead to a dysbiosis of the microbial community, harming the host? And ii) do the intestinal microbiota contribute to the states and consequences of poor nutrition, similar to that which has been described for undernourished mice^26^? In order to unravel these critical questions, a study with repeated sampling over a period of undernutrition and refeeding would be necessary. Also, stool sample transplantations into gnotobiotic mice (and/or pigs where the digestive tract resembles more the situation in humans) would help to further understand the AN patient host-microbe biology. Additionally, to gain insights on gut microbiota and their pathways, there should be special focus on measurements of other proteolytic fermentation products in the gut, which may play a key role in the AN pathology and possibly also in other forms of undernutrition.

In contrast to carbohydrate digestion in the large bowel, the breakdown of proteins by gut microbiota has been largely ignored by the research community^18^ and should be the focus of future studies. Furthermore, a large long-term prospective study would be important in order to unravel, whether the intestinal microbiota contributes to AN similarly to the results in kwashiorkor^19^. Finally, carefully planned and conducted gut microbiota-targeted intervention studies in the context of well established AN inpatient and outpatient therapies may be a promising concept to support weight recovery and to improve psychological and gastrointestinal symptoms. Since only 50% of the patients recover fully in the long-term, additional therapeutic approaches are needed. We suggest that butyrate producing taxa (e.g. *Roseburia spp.*) could be interesting candidates for such intervention studies. Similar to *Faecalibacterium prausnitzii*, a depletion of *Rosebura spp*. has been also linked to other intestinal diseases such as IBD^45^.

We conclude that i) the gut microbiota of AN patients is perturbed in comparison to NW participants and ii) these perturbations did not recover after weight gain and/or normalisation of eating behaviour in addition to several gastrointestinal symptoms. Strikingly, the microbial perturbations were accompanied by increased concentrations of BCFA, being markers for protein fermentation. Therefore, our insights into the microbial perturbations in AN provide a basis for new leads to modulate the gut microbiota in order to influence nutritional status and improve long-term outcomes and/or side effects such as psychological diseases and gastrointestinal symptoms in these patients and possibly also in other forms of undernutrition.

## Methods

### Ethics and Registration of the Study

All experiments were performed in accordance with relevant guidelines and regulations. The study protocol was approved by the Ethics Committee of the University Hospital Tübingen, Germany (No. 429/2011BO2) and is registered at the German Clinical Trials Register (DRKS00005124). Participants were informed about the study purpose during the admission interview and were asked to provide written consent prior inclusion. In case of minors (<18 years of age) the parents were contacted by telephone and had to provide written informed consent.

### Design and population of the study

#### AN patients

AN patients were recruited within the context of an impatient treatment program at Schön Klinik Roseneck, Prien, Germany, aiming at increasing body weight and normalizing eating habits in patients with a primary diagnosis of AN. Within the first week upon admission, female AN patients of all subtypes and aged between 14 and 39 years with a BMI <18 kg/m^2^ in adults or <10% of the expected weight in adolescents were recruited and primarily included in the study. Patients having taken antibiotics within the last eight weeks, or with severe somatic diseases including renal failure and liver dysfunction, or with limited German verbal skills or unable to understand the instructions and perform stool sampling were excluded.

#### NW participants

NW participants matched for age and gender were students, employees, or children of employees from the University of Tübingen, Germany. The exclusion criteria were the same as those for AN patients, and in addition, a BMI <18 kg/m^2^ in adults or <10% of the expected weight in adolescents, respectively was an exclusion criterion.

#### Sampling

Stool samples of AN patients were collected as soon as possible after the beginning of their inpatient stay. Patients were provided with a stool sampling kit and instructed to auto-collect their stool by placing a stool collector (Süsse Labortechnik, Gudensberg, Germany) into the toilet before defaecation. Next, they sampled stool with a spoon from eight different sites of the whole faeces into two leak proof receptacles with screw cap (Roth, Karlsruhe, Germany). Immediately, upon defaecation, patients delivered their stool samples to the collection point for human specimens at the hospital where one of the receptacles was instantly frozen at −80 °C and the second receptacle was mailed directly to the Institute of Microecology, Herborn, Germany, where a blind analysis of the short chain fatty acids (SCFA) was conducted (see section SCFA). Controls were instructed to auto-collect their faecal sample at home, using the sampling kit and following the same instructions, and to immediately contact staff from the University Hospital of Tübingen upon defaecation. University staff picked up samples straight away, transported the samples between cool packs (stored ahead at +4 °C) and subsequently stored the samples at −80 °C upon arrival. The median time to freezing was 0:45 [0.15–1:55] hours.

### Techniques and analysis

#### Instruments for assessing nutrition intake and statistics

ANT1: After study inclusion, AN patients were asked by a trained interviewer to report their 24 hour food intake of the day before admission to the hospital. If this was not a representative day of food intake, they were asked to report the food intake of a typical 24 hour day (without having a binge episode if AN patients with a binge/purging type were interviewed). Additionally, the frequencies but not the quantities of a validated food frequency questionnaire (FFQ)^46^ were provided by the patients. Quantities were not requested since the instrument was not validated for extremely small quantities.

ANT2: In the hospital, the patients received a standard diet designed for AN. Depending on the progress of weight gain, AN patients received diet according to one of four diet plans. The standard diet consists of 2000 kcal/d, the diet+ consists of 2400 kcal/d, the diet++ consists of 2800 kcal/d and the diet+++ consist of 3050 kcal/d. In this diet, 15% of the energy intake corresponds to protein, 45% to fat and 40% to carbohydrates. Ahead of T2 sampling, a 24 h recall was conducted with the patients by a trained interviewer. Again, the frequencies of the FFQ were collected.

NW: The multiple source method (MSM) was used to estimate the usual food intake for NW individuals by combining short-term and long-term measurements of dietary intake^47^. Therefore, participants recorded their food intake for 24 hours on two non-consecutive days. Additionally, the frequencies and quantities of the FFQ were filled in by the participants, serving as a long-term measurement of dietary intake^46^. Next, the MSM algorithm was applied to calculate the usual intake of calories, fat, carbohydrates and protein. As tool the program package of the MSM website was used (https://nugo.dife.de/msm). This method was applied in order to receive as representative as possible data on nutrition intake from NW participants for comparison with AN patients.

The data were non-normally distributed. Differences between AN patients before and after weight gain and between AN patients and NW participants were compared using the Wilcoxon signed-rank test and Mann-Whitney-U-test, respectively. The p-values of food group consumption frequencies were FDR-adjusted. For all other nutrition data a p < 0.01 was considered as statistically significant.

#### Instrument for assessing gastrointestinal symptoms and statistics

The validated gastro-questionnaire^48^ was used to assess the frequency and severity of 27 gastrointestinal symptom items. This questionnaire has been used for repeated dimensional measurements^48^. AN patients (at T1) and NW participants were asked to rate all gastrointestinal symptoms that had occurred at least 25% of the time (3 months continuously or in intervals) within the preceding 12 months. At T2, the patients were asked to report all gastrointestinal symptoms that had occurred during the last 4 weeks of their inpatient stay. Each question consisted of two parts. Part one asked for the frequency of the symptoms (rated between 0 = “not at all” to 3 = “most of the time”). Part two asked to rate the symptom severity between 1 = “no distress” 5 = “severe distress”). In order to calculate a variable providing information on both, ratings for frequency and severity, for every single symptom, were multiplied resulting in a Severity Score that varied between 0 and 15 per symptom. Subsequently, the sum of scores for upper GI-symptoms (UGIS) and lower GI-symptoms (LGIS) were calculated. The UGIS-score included the following symptoms: Sensation of lump in the throat (independently from meals), regurgitation of food, difficulty swallowing, nausea, vomiting, heartburn (changing after meals and relieved by antacids), chest pain, abdominal fullness, abdominal bloating, and feeling of abdominal distension (10 items). The LGIS-score included abdominal pain, bowel noises, stools very often (>3 defaecations daily), stools very rarely (<3 defaecations per week), stool urgency, faecal soiling, frequent changing of stool consistency, loose or watery stools, hard or lumpy stools, straining during a bowl movement, a feeling of incomplete evacuation, frequent passing of gas, and pain in the rectum (13 items). Finally, the sum of the scores was divided by the number of included items in order to allow comparisons between the UGIS-score and LGIS-score. The scores were non-normally distributed. Differences in the scores between AN patients before and after weight gain and between AN patients and NW participants were compared using the Wilcoxon signed-rank test and Mann-Whitney-U-test respectively. Spearman correlations between ANT1 versus NW participants and between ANT2 and NW participants, respectively were used to analyse differences for single GI severity scores; differences within AN, were analysed with Wilcoxon-signed rank test. The p-values of all analyses were FDR-adjusted.

#### Techniques for assessing microbial analysis

For DNA isolation and amplicon library preparation, the samples were selected randomly in order to prevent batch effects. The latter procedure and sequencing was performed by blinded investigators. Subsequently, the mapping file was provided for data analysis.

##### DNA isolation

PSP lysis buffer (Stratec Molecular, Berlin, Germany) was added to a sterile vial containing 0.5 g of 0.1 mm zirconia/silica beads and 4 3.0–3.5 mm glass beads (BioSpec, Bartlesville, USA). 200 mg of frozen stool aliquots were added to the vials. The samples were homogenized in a MagNA Lyser instrument (Roche, Basel, Switzerland) in three cycles of 1 min. at a speed of 5500 rpm. Samples were kept on ice for one minute in between cycles. DNA isolation was continued using the PSP Spin Stool Kit (Stratec Molecular, Berlin, Germany) according to the manufacturers’ instructions. DNA was finally eluted in 200 μl elution buffer.

##### Sequencing

Amplicon libraries and sequencing was performed according to previously published protocols^49^. Briefly, the V4 region of the 16S rRNA gene was PCR amplified from each DNA sample in triplicate using the 515f/806r primer pair described previously^49^. Pooled amplicons from the triplicate reactions were purified using AMPure XP purification (Agencourt, Massachusetts, USA) according to the manufacturer’s instructions and eluted in 25 μl 1× low TE (10 mM Tris-HCl, 0.1 mM EDTA, pH 8.0) and subsequently quantified by Quant-iT PicoGreen dsDNA reagent kit (Invitrogen, New York, USA) using a Victor3 Multilabel Counter (Perkin Elmer, Waltham, USA). Amplicons were mixed in equimolar concentrations, to ensure equal representation of each sample, and sequenced on an Illumina MiSeq instrument.

#### Data analysis and statistics

The V4 16S rDNA bacterial sequences that were used in this paper have been submitted to the EMBL databases under accession No. PRJEB11199.

Filtering, denoising, removing of chimeric sequences and clustering of sequences in Operational Taxonomic Units (OTUs) at 97% similarity was conducted using the LotuS (version 1.39) pipeline^50^. First, sequences with an average quality below 27, read length below 170 bases, one or more ambiguous bases, or containing homopolymer stretches of over 8 bases were discarded for further analysis. Retained sequences were chimera filtered and clustered into OTUs with UPARSE^51^. Taxonomic annotation of OTUs was derived from RDP naïve bayes classifier annotations (minimum acceptance confidence set at 0.8)^52^.

Singletons and OTUs present in less than 3% of the samples were removed. Downstream analyses were conducted using QIIME version 1.8.1^53^ and R version 3.1.3.

##### Taxonomy, alpha and beta diversity, microbial core

Differences in the relative proportion of bacterial phyla between AN patients before and after weight gain and between AN patients and NW participants were compared using the Wilcoxon signed-rank test and Mann-Whitney-U-test respectively. To examine which genera significantly increased or decreased in AN patients during the intervention, relative abundances were compared using the Wilcoxon signed rank test. Spearman rank’s correlation were calculated to evaluate differences in the relative abundance of genera between AN patients and NW participants. All p-values were controlled for multiple testing using the False Discovery Rate (FDR).

The following metrics of species richness and diversity within communities (alpha-diversity) were determined: observed OTUs (observed richness), Chao1 index (estimated richness), and Shannon diversity index. Alpha diversity metrics in AN patients before and after weight gain were compared by paired t-tests, whereas unpaired t-tests were used to compare alpha diversity metrics of AN patients to NW participants. Subsequently, the p-values were Bonferroni adjusted.

Beta-diversity, or diversity shared across samples was determined by the unweighted and weighted UniFrac distance^54^ and Bray-Curtis dissimilarity (BC) at a rarefaction depth of 26,190 seq/sample. Clustering of samples was visualized using Principal Coordinate analysis (PCoA). Single-factor ANOVA followed by Bonferroni post-hoc analysis for BC and the Kruskal-Wallis-test for unweighted UniFrac distance, respectively, were used to compare the beta-diversity within AN subjects (mean distance between sample at T1 and T2 per subject) to the beta-diversity between AN subjects at T1, AN subjects at T2 and NW participants respectively. Only p-values < 0.001 were considered as statistically significant.

To examine differences in the microbial core of AN patients before and after weight gain and NW participants, the number of OTUs present in a fraction of subjects per group ranging from 75 to 100% was calculated. We subsequently assessed which OTUs were shared in the core, defined as 90% of samples that an OTU must be observed in to be considered part of the core, of AN patients before and after weight gain and NW participants.

##### Enterotyping

Enterotype analyses were performed as described previously by Armougham and colleagues^55^. Jensen–Shannon (JD) divergence, Bray-Curtis (BC), and Euclidian distances were calculated for the genus-level relative abundance profiles. We used the R^56^ package “vegan: Community ecology”, version 2.2-1 by Oksanen *et al*. from 2011 for calculating the Bray-Curtis and Euclidian distances.

To cluster the samples based on these distance metrics, we used the Partitioning Around Medoids (PAM) clustering algorithm in the R^56^ package “Cluster analysis basics and extensions”, version 2.0.1 ed. by Maechler *et al*. from 2012. The optimal number of clusters was chosen based on Calinski-Harabasz (CH) index and validated by the silhouette index^57^ using the R^56^ package ‘clusterSim’.

For each of the distance metrics, an optimal number of 2 clusters were identified based on the CH index, which was confirmed by the silhouette index although clustering was moderate (SI ranging from 0.41 for JD to 0.59 for Euclidian).

Subsequent visualization and identification of relevant taxa was conducted for analyses based on BC distance. Between-class analysis (BCA) was performed to plot the samples using the R^56^ package “Analysis of Ecological Data: Exploratory and Euclidean Methods in Environmental Sciences” version 1.7.2. by Dray *et al*. from 2015. The similarity percentage analysis (SIMPER)^58^ was used to identify taxa contributing to similarity within- and dissimilarity between groups.

##### distance-based Redundancy Analysis (db-RDA)

Distance-based redundancy analysis (db-RDA), a constrained extension of PCoA, was performed with the capscale function in vegan. To determine to what extend anorexia type (restrictive, binge eating/purging), age, and intake of total energy, fibre, protein and fat contribute in explaining the microbial community structure, we used variance partitioning with distance-based redundancy analysis (db-RDA) of Bray-Curtis distances of all samples of AN patients at baseline.

We also examined the extent to which disease status explained the microbial community structure using db-RDA of BC distances of baselines samples from both NW participants and AN patients.

#### Determination of Short Chain Fatty Acids (SCFA) and statistics

SCFA were analysed in stool samples using gas chromatography. The samples were weighed (~200 mg dry matter) and 10-fold diluted with physical NaCl (1, 8 ml). Following vortexing (2 min,Vortexer MS 3 basic, IKA-Werke GmbH&Co.KG, Staufen, Germany), an aliquot of 200 μl was mixed with 0, 36 M HClO4 (280 μl) and 1 M NaOH (270 μl). The solution was lyophilised at −35 °C (Alpha 1–4 LSC, CHRIST, Osterode am Harz, Germany). The lyophilisate obtained was dissolved in 100 μl 5 M HCOOH and 400 μl Aceton and centrifuged (5 min at 4000× g, RT; Pico 17, Thermo Electron LED GmbH, Langenselbold, Germany). Concentrations of the SCFA were determined in the supernatant using a GC-2010 Plus gas chromatograph (Shimadzu Deutschland GmbH, Duisburg, Germany) equipped with a flame ionization detection with a thin-film capillary column Stabilwax®-DA 30 m × 0, 25 mm × 0, 5 μm (Restek, Bad Homburg, Germany). The samples were spread out by split injection using the auto-sampler AOC-20s/I (Shimadzu Deutschland GmbH). GCsolution Chromatography Data System (Shimadzu Deutschland GmbH) was used for data processing. For determination of the SCFA an external standard (SupelcoTM WSFA-1 Mix, Supelco Sigma-Aldrich Co., Bellefonte PA) was used. The data were non-normally distributed. Differences between AN patients before and after weight gain and between AN patients and NW participants were compared using the Wilcoxon signed-rank test and Mann-Whitney-U-test, respectively. Spearman correlations were used to analyse the relationships between SCFA and taxa at genus level present in at least 25% of all participants. The p-values of all analyses were FDR-adjusted.

## Additional Information

**How to cite this article**: Mack, I. *et al*. Weight gain in anorexia nervosa does not ameliorate the faecal microbiota, branched chain fatty acid profiles, and gastrointestinal complaints. *Sci. Rep.* **6**, 26752; doi: 10.1038/srep26752 (2016).

## Supplementary Material

Supplementary Information

## Figures and Tables

**Table 1:** Short chain fatty acids (SCFA) in anorexia nervosa patients (AN) before (T1) and after (T2) weight gain compared to normal-weight participants (NW).

A	SCFA [μmol/g]			
	ANT1	ANT2	NW	Group differences
	Median [IQR]	Median [IQR]	Median [IQR]	
Total SCFA	39.5 [27.1–56.8]	43.8 [26.8–68.6]	43.5 [29.8–65.7]	n.s.
Acetate	25.6 [18.5–35.8]	29.3 [16.6–43.9]	29.9 [20.5–41.5]	n.s.
Butyrate	4.4 [2.5–6.8]	6.1 [3.1–10.4]	5.4 [3.4–11.8]	n.s.
Propionate	5.7 [3.4–7.6]	6.8 [4.3–10.2]	6.7 [4.1–10.3]	n.s.
Valerate	1.2 [0.9–1.5]	1.1 [0.6–1.6]	0.7 [0.4–1.2]	ANT1-NW***, ANT2-NW*
Total BCFA	1.9 [1.5–2.3]	1.7 [1.2–2.4]	1.3 [0.8–2.1]	ANT1-NW*, ANT2-NW*
Iso-valerate	0.9 [0.7–1.2]	0.9 [0.6–1.4]	0.7 [0.4–1.2]	n.s.
Iso-butyrate	0.9 [0.7–1.2]	0.9 [0.5–1.1]	0.6 [0.3–0.9]	ANT1-NW*, ANT2-NW*
**B**	**Relative proportion of specific SCFA in relation to total SCFA [%]**			
	**ANT1**	**ANT2**	**NW**	
	**Median [IQR]**	**Median [IQR]**	**Median [IQR]**	**Group differences**
Acetate	67.3 [63.9–69.6]	65.2 [59.7–70.4]	66.6 [61–70.4]	n.s.
Butyrate	11.0 [7.7–12.9]	13.9 [10.4–19.5]	12.7 [9.7–17.7]	ANT1-ANT2**, ANT1-NW*
Propionate	12.9 [11.6–15.7]	13.9 [11.6–16.1]	14.9 [12.7–17.4]	n.s.
Valerate	3.0 [2.1–4.7]	2.5 [1.8–4.0]	1.6 [1.1–2.4]	ANT1-NW***, ANT2-NW**
Total BCFA	4.7 [3.3–6.3]	4.0 [2.9–4.9]	3.0 [2.3–3.8]	ANT1-NW***, ANT2-NW**

SCFA concentrations (**A**) and the relative proportion of specific SCFA in relation to total SCFA (**B**) are presented. The p-values were false discovery rate (FDR) adjusted. A FDR < 0.05 was considered as statistically significant. *Indicates a FDR < 0.05 and **a FDR < 0.01. BCFA, branched chain fatty acids.
