# Supplementary material for: Weight gain in anorexia nervosa does not ameliorate the faecal microbiota, branched chain fatty acid profiles, and gastrointestinal complaints
Source: Sci Rep. 2016 May 27;6:26752. doi: 10.1038/srep26752 (PMC4882621; doi:10.1038/srep26752)
Supplement: Supplementary Information [file srep26752-s1.pdf]

Supplements 1 to 4

Weight gain in anorexia nervosa does not ameliorate the faecal microbiota, branched chain fatty acid profiles, and gastrointestinal complaints

Isabelle Mack, Ph.D.[1], Ulrich Cuntz, M.D.[2], Claudia Grämer, Dipl. Nutrionist[2], Sabrina Niedermaier, Dipl. Nutrionist[2], Charlotte Pohl, cand. M.D.[1], Anderas Schwiertz, Ph.D.[3], Kurt Zimmermann, Ph.D [4], Stephan Zipfel, M.D.[1], Paul Enck, Ph.D.[1], John Penders, Ph.D.[5]

[1]Department of Psychosomatic Medicine and Psychotherapy, University Medical Hospital, Tübingen, Germany; [2]Klinik Roseneck, Center for Behavioral Medicine, Prien, Germany; [3]Institute of Microecology, Herborn, Germany; [4]SymbioPharm GmbH, Herborn, Germany; [5]Maastricht University Medical Center, NUTRIM School for Nutrition and Translational Research in Metabolism, Department of Medical Microbiology, Maastricht, The Netherlands

Supplement 1: Food consumption frequencies of anorexia nervosa patients (AN) before (T1) and after (T2) weight gain in comparison to normal-weight participants (NW).

	ANT1			ANT2			NW			FDR	FDR	FDR
**Frequency per month**	**P25**	**Median**	**P75**	**P25**	**Median**	**P75**	**P25**	**Median**	**P75**	**ANT1 vs. NW**	**ANT2 vs. NW**	**ANT1 vs. ANT2**
Milk	3	28	56	1	6	28	14	28	84		0.002	
Sugar-sweetened beverages	0	0	3	0	0	1	1	3	6	<0.001	<0.001	
Calorie-reduced beverages	0	6	70	0	1	6	0	0	3	<0.001		0.001
Fruit juice	0	1	6	0	1	3	5	14	35	<0.001	<0.001	
Vegetable juice	0	0	0	0	0	0	0	0	0			
Water	84	168	168	84	126	168	116	126	168			
Fruit and herbal tea	14	56	126	14	28	56	1	6	24	<0.001	<0.001	
Black tea	0	0	14	0	0	3	0	1	3			
Coffee	3	28	56	3	28	56	3	14	56			
Beer	0	0	1	0	0	0	0	1	6	<0.001	<0.001	0.037
Alcohol-free beer	0	0	0	0	0	0	0	0	0			
Wine, sparkling wine	0	0	3	0	0	1	1	3	6	<0.001	<0.001	
Cocktails	0	0	1	0	0	0	0	1	3	0.010		
Schnapps	0	0	1	0	0	0	0	1	1	0.029	0.004	
Corn flakes, cocao crispies	0	0	1	0	0	1	0	0	3			
Muesli, cereals	0	1	6	0	3	14	1	3	6			
Whole-meal bread or rolls	3	28	28	56	56	56	5	14	22		<0.001	<0.001
Brown bread	0	1	14	1	22	28	1	6	6		0.017	0.042
White bread, toast, rolls	0	3	14	6	22	28	3	6	14		0.004	0.011
Butter	0	3	25	56	56	56	5	18	56	0.01	<0.001	<0.001
Cream cheese	1	6	28	6	14	28	1	6	14		<0.001	
Cheese	1	6	25	14	28	56	5	6	14		<0.001	<0.001
Yoghurt, quark	3	14	28	14	22	28	3	6	14		<0.001	
Honey, marmelade, syrup	0	6	28	28	28	28	3	3	14		<0.001	<0.001
Hazelnut-chocolate spread	0	0	1	0	3	6	0	1	6	0.003		0.049
Eggs	0	1	3	3	6	6	2	3	6	<0.001		0.002
Poultry	0	3	6	0	3	6	1	3	6			
Doner kebab, hamburger	0	0	0	0	0	0	0	1	3	<0.001	<0.001	
Bratwurst/sausage	0	0	0	0	0	0	0	1	1	<0.001	<0.001	
Meat	0	1	3	0	6	6	1	3	6	0.044		0.008
Sausage, cold cuts	0	0	1	0	1	3	0	4	14	<0.001	0.004	
Ham	0	1	6	0	6	14	0	3	6			
Cold fish	0	1	3	0	3	6	0	0	3			
Cooked fish	0	3	6	3	6	6	0	2	3		<0.001	
Fresh fruit	6	28	56	14	14	28	14	28	56			
Cooked fruit	0	0	3	0	3	6	0	1	3		0.002	
Raw vegetables	6	28	56	14	14	28	14	22	28			0.011
Pulses	0	3	6	1	3	6	1	3	3			
Cooked vegetables	6	22	28	14	22	28	3	6	14	0.019	<0.001	
Pasta	1	3	6	6	6	6	6	6	14	<0.001		0.049
Rice	0	3	6	6	6	14	3	3	6		<0.001	0.019
Cooked potatoes	1	3	6	6	6	14	3	3	6		<0.001	0.001
Fried potatoes	0	0	1	0	1	3	0	1	1			0.002
Chips	0	0	0	0	0	0	1	1	3	<0.001	<0.001	
Pizza	0	0	1	0	1	3	1	1	3	<0.001	0.015	
Cake, muffins	0	1	10	3	6	14	3	3	6		0.011	
Biscuits	0	0	6	0	3	6	1	3	6			
Chocolate	0	1	14	3	6	14	3	6	14	0.003		
Sweets	0	6	28	0	3	18	1	3	14			
Ice cream	0	1	3	1	3	6	1	3	6	0.009		
Crisps	0	0	0	0	0	0	0	1	3	<0.001	<0.001	
Salty bicuits, salt sticks	0	0	3	0	0	1	0	1	1		0.008	
Nuts	0	1	4	0	1	3	0	2	3		0.055	

Legend: The median, P25 and P75 values for the food consumption frequencies within the last 28 days are presented. All p-values were false discovery rate (FDR)-adjusted. A FDR< 0.05 was considered as statistically significant. Annotation: The frequencies provide no information on the portion sizes consumed.

Supplement 2: Severity scores for gastrointestinal symptoms (GIS) in anorexia nervosa patients (AN) before (T1) and after (T2) weight gain.

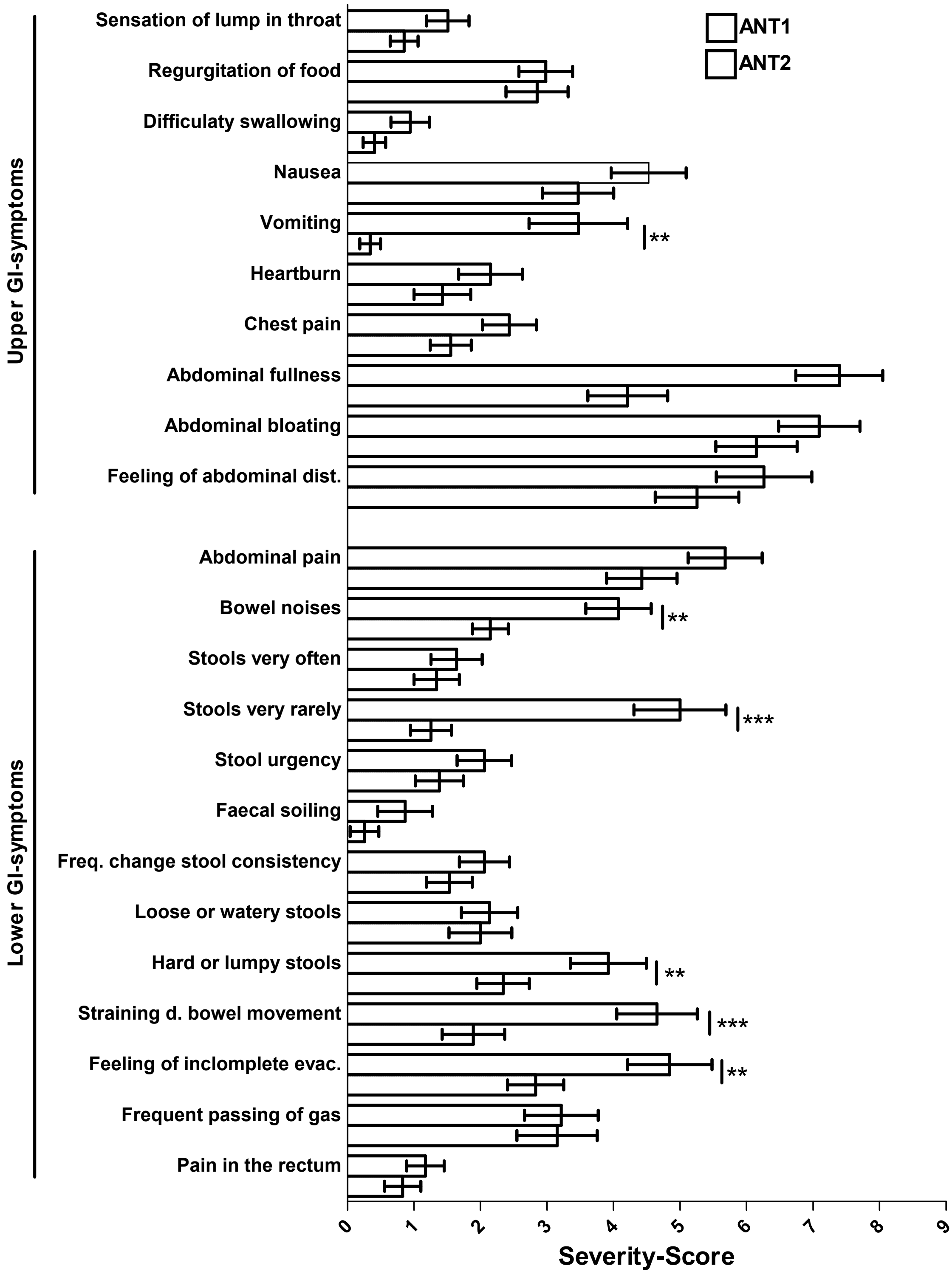

Legend: All differences of GIS between T1 and T2 in AN are given as mean±SE for clarity, although the data are not normally distributed. All p-values were false discovery rate (FDR)-adjusted. A FDR <0.05 was considered as statistically significant. ** indicates a FDR <0.01 and *** indicates a FDR <0.001.

Supplement 3: Correlations between SCFA and taxa at genus level for anorexia nervosa patients (AN) before (T1) and after (T2) weight gain and normal weight participants (NW).

Phylum_Genus	Acetate			Butyrate			Propionate			Valerate			Isovalerate			Isobutyrate		
	ANT1	ANT2	NW	ANT1	ANT2	NW	ANT1	ANT2	NW	ANT1	ANT2	NW	ANT1	ANT2	NW	ANT1	ANT2	NW
Actinobacteria_Bifidobacterium								-0,314										
Actinobacteria_Collinsella										***0,376***			0,304					
Actinobacteria_Eggerthella	-0,296	-0,311		-0,303	***-0,364***		-0,312				***-0,392***		-0,372	***-0,458***			-0,394	
Actinobacteria_Gordonibacter		-0,309			***-0,406***			***-0,422***										
Bacteroidetes_Bacteroides											***-0,418***						-0,37	
Bacteroidetes_Barnesiella									-0,275									
Bacteroidetes_Butyricimonas																0,312		0,333
Bacteroidetes_Paraprevotella									0,303									
Bacteroidetes_Prevotella											0,32							
Bacteroidetes_Alistipes										0,281								
Firmicutes_Lactococcus																		
Firmicutes_Streptococcus			0,298									***0,37***			0,338			***0,39***
Firmicutes_Clostridium_sensu_stricto		***0,417***			0,313			***0,394***		0,277	***0,451***			***0,426***			***0,444***	
Firmicutes_Blautia				0,29														
Firmicutes_Clostridium_XIVb																		
Firmicutes_Coprococcus				**0,429**											0,315			
Firmicutes_Dorea			0,274						0,354		***0,439***	***0,398***	**0,459**	0,324	0,326		***0,452***	***0,404***
Firmicutes_Lachnospiracea_incertae_sedis																		
Firmicutes_Roseburia	0,366	***0,425***		**0,453**	**0,535**	0,38	0,308	**0,486**						0,308				
Firmicutes_Clostridium_XI		0,325									***0,42***			***0,427***			***0,442***	
Firmicutes_Anaerotruncus	-0,346			**-0,435**			**-0,425**											
Firmicutes_Butyricicoccus		***0,407***			0,3290									0,324				
Firmicutes_Clostridium_IV							-0,294										0,328	
Firmicutes_Faecalibacterium		***0,375***			**0,451**			***0,405***										
Firmicutes_Flavonifractor				-0,306	-0,313								-0,373					
Firmicutes_Pseudoflavonifractor							-0,272											
Firmicutes_Ruminococcus					0,312													
Firmicutes_Sporobacter	-0,284						***-0,351***			0,281								
Firmicutes_Subdoligranulum																		
Firmicutes_Clostridium_XVIII										***-0,364***			-0,334					
Firmicutes_Holdemania																		
Firmicutes_Turicibacter				-0,282			-0,277				***0,384***			0,336			0,365	
Firmicutes_Phascolarctobacterium				***0,337***			0,277											
Firmicutes_Dialister																-0,273		
Proteobacteria_Oxalobacter																		
Proteobacteria_Parasutterella			-0,304			-0,271			-0,288						-0,271			
Proteobacteria_Sutterella																		
Proteobacteria_Bilophila		***-0,379***			**-0,498**			***-0,434***							0,298			0,289
Proteobacteria_EscherichiaShigella			0,315						0,378			***0,423***			0,345			**0,449**
Verrucomicrobia_Akkermansia							-0,286											

Legend: Spearman correlations between SCFA and bacterial genera that were present in at least 25% of the participants were conducted for all three groups separately. The p-values were false discovery rate (FDR) adjusted. A FDR<0.2 was considered as statistically significant. The table shows the correlation coefficients in bold if FDR<0.05, in bold italics if FDR>0.05 but <0.02 and in normal font if they did not withstand FDR adjustment.

Supplement 4: The role of laxative use in anorexia nervosa patients (AN) on the intestinal microbiota.

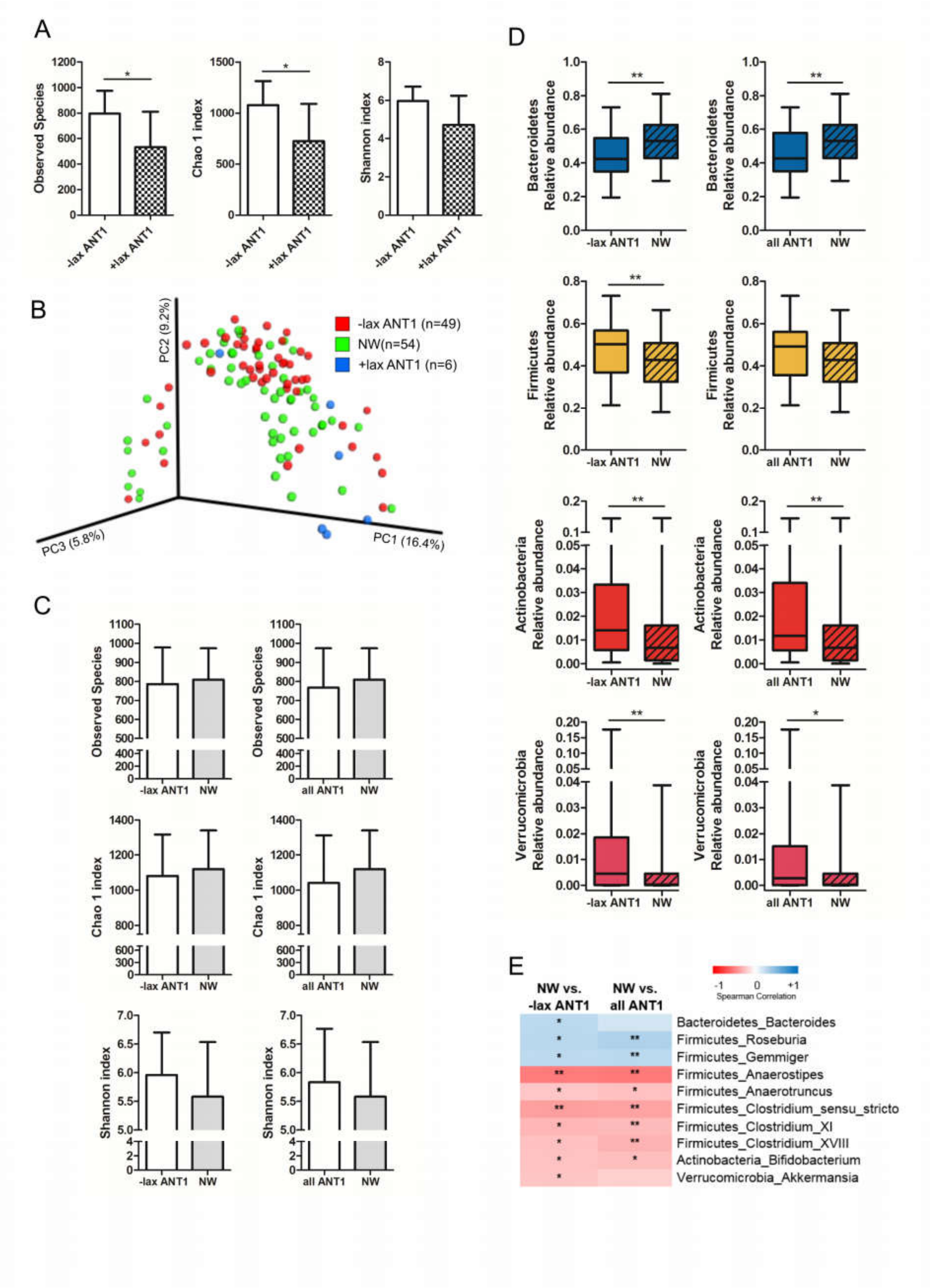

Legend (Suppl.4) **A:** Alpha diversity metrics are presented for AN patients without (-lax) and with a history (+lax) of laxative use before their inpatient stay (T1). **B:** Principal Coordinate Analysis (PCoA) of Bray-Curtis dissimilarities coloured according to –lax ANT1 (green), +lax ANT1 (blue) and normal-weight participants (NW; green). **C-E:** Sensitivity analysis: Alpha diversity metrics (**C**) and taxonomy at phylum (**D**) and genus level (**E**) are presented for NW versus ANT1 participants without a history of laxative use (-lax ANT1). In order to better interpret these sensitivity analysis, the data for NW versus ANT1 including patients with a history of laxative use (all AN, data from figures 3 and 5) are shown for comparison. P-values were adjusted for multiple testing (see methods). * indicates $p < 0.05$, except for D+E where after false discovery rate (FDR) adjustment a FDR <0.15 was considered as statistically significant in order to account for the beta error and where * indicates a FDR<0.15 and ** a FDR <0.05.
